# Correction: Integrative genomic analysis of the human immune response to influenza vaccination

**DOI:** 10.7554/eLife.18898

**Published:** 2016-08-30

**Authors:** Luis M Franco, Kristine L Bucasas, Janet M Wells, Diane Niño, Xueqing Wang, Gladys E Zapata, Nancy Arden, Alexander Renwick, Peng Yu, John M Quarles, Molly S Bray, Robert B Couch, John W Belmont, Chad A Shaw

Franco LM, Bucasas KL, Wells JM, Niño D, Wang X, Zapata GE, Arden N, Renwick A, Yu P, Quarles JM, Bray MS, Couch RB, Belmont JW, Shaw CA. 2013. Integrative genomic analysis of the human immune response to influenza vaccination. *eLife* **2**:e00299. doi: 10.7554/eLife.00299.

Published July 16, 2015

In our manuscript concerning integrative genomic analysis of the response to influenza vaccination in humans, we included a section concerning perturbation eQTLs. Perturbation eQTLs are associations of gene expression with genotype that may change in magnitude, direction, or only appear after exposure of individuals to experimentally modifiable covariates or variable environmental conditions. In the case of our study, influenza vaccination serves as a perturbation exposure, and gene expression profiling was performed serially on subjects before and after the vaccination. This design permits the investigation of perturbation eQTLs.

Our paper presented an expository of a perturbation eQTL using the gene *NECAB2* (Figure 2A), as well as more global analysis of perturbation eQTLs characteristics in our data (Figure 2B). In error, we presented two identical Manhattan plot graphics for *NECAB2* in Figure 2A, both from male subjects instead of graphics for the distinct male and female cohorts in our study. Upon review, the male plot strongly shows the induction of an association signal after exposure, but the female cohort shows an association signal at baseline. Therefore, *NECAB2* does not replicate the pattern that would be expected of a perturbation eQTL. Nevertheless, perturbation eQTLs are potential contributors to association patterns in our study. This was illustrated in our global analysis, which showed an increase in the magnitude of the genotype effect (R2g), explained by an increase in beta without evidence of decrease in the mean within genotype variance (presented in Figure 2B). Our objective in presenting *NECAB2* was to provide a motivating example, and *NECAB2* as a perturbation was not otherwise important to the conclusions in our work. The correct pair of Manhattan plots for *NECAB2* has been included here (Correction figure 1).

**Correction figure 1.**
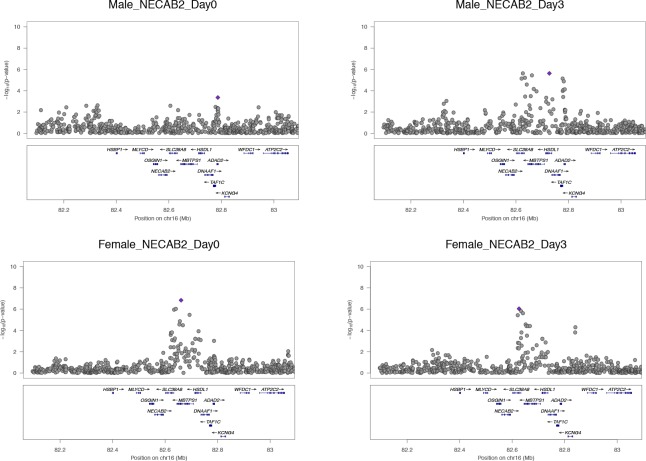
*NECAB2*. The locus shows eQTL activation in the male cohort but not in the female cohort. This figure addresses the error in our original Figure 2A.

To clarify the potential contribution of perturbation eQTLs, we present additional expository examples of the genes *ADCY3* (Correction figure 2) and *DISC1* (Correction figure 3). These genes present a replicated perturbation eQTL pattern in both the male and female data.

**Correction figure 2.**
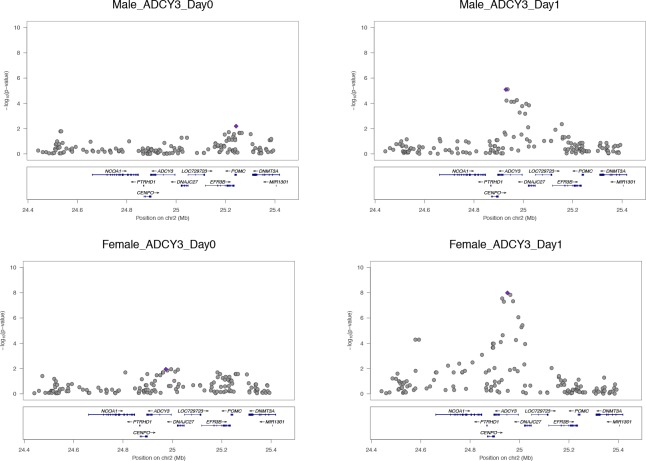
*ADCY3*. A replicated perturbation eQTL locus activated on Day 1 in both cohorts.

**Correction figure 3.**
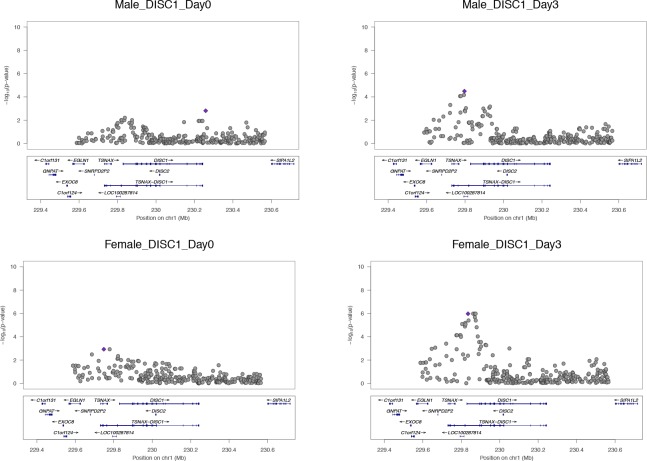
*DISC1*. A replicated perturbation eQTL locus activated on Day 3 in both cohorts.

We appreciate the opportunity to correct the error made in presenting the graphics of *NECAB2*. The corrected version of Figure 2 is shown below:

**Figure:**
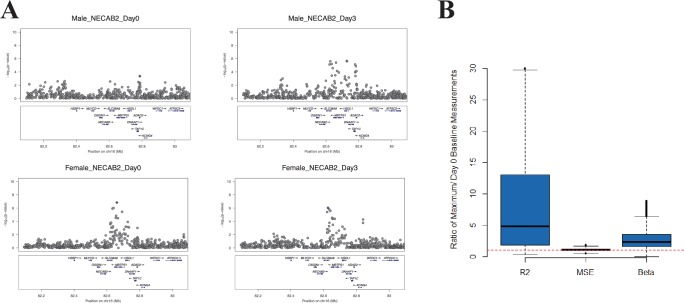

The originally published Figure 2 is also shown for reference:

**Figure:**
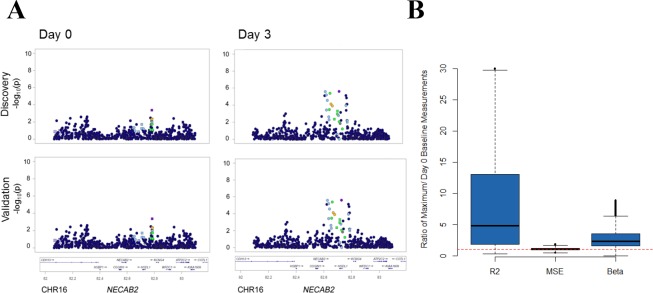

More research in this area is warranted, and we hope other studies will examine the potential of this distinctive form of gene–environment interaction.

The article has been corrected accordingly.

